# Prolonged PSA stabilization and overall survival following sipuleucel-T monotherapy in metastatic castration-resistant prostate cancer patients

**DOI:** 10.1038/s41391-019-0144-3

**Published:** 2019-04-12

**Authors:** Eda K. Holl, Megan A. McNamara, Patrick Healy, Monika Anand, Raoul S. Concepcion, Coleman D. Breland, Igor Dumbudze, Ron Tutrone, Neal Shore, Andrew J. Armstrong, Michael Harrison, Joe A. Wallace, Yuan Wu, Daniel J. George

**Affiliations:** 1 Department of Surgery, Duke University Medical Center, Durham, NC 27710 USA; 2 Division of Medical Oncology, Departments of Medicine, Duke Prostate and Urologic Cancer Center, Duke University and the Duke Cancer Institute, Durham, NC USA; 3 Urology Associates, P.C. 2801 Charlotte Ave., Nashville, TN 37209 USA; 4 TriState Urologic Services PSC, Inc., The Urology Group, 2000 Joseph E. Sanker Blvd., Cincinnati, OH 45212 USA; 5 Chesapeake Urology Associates, 6820 Hospital Dr., Suite 210, Baltimore, MD 21237 USA; 6 Carolina Urologic Research Center, 823 82nd Pkwy., Ste. B, Myrtle Beach, SC 29572 USA; 7 Division of Medical Oncology, Department of Pharmacology and Cancer Biology, Duke Prostate and Urologic Cancer Center, Duke University and the Duke Cancer Institute, Durham, NC USA

**Keywords:** Prostate cancer, Cancer therapy

## Abstract

**Background:**

Sipuleucel-T is an autologous cellular immunotherapy that is FDA approved for the treatment of asymptomatic or minimally symptomatic metastatic castrate-resistant prostate cancer (mCRPC). The IMPACT registry trial demonstrated a 4.1 month survival benefit, but not a consistent PSA response or improvement in progression-free survival. Based upon several factors, including this lack of objective treatment response, sipuleucel-T has been under-utilized in this patient population, despite current NCCN recommendations.

**Methods:**

In order to explore if delayed treatment response occurs in a subset of patients, we performed a single institutional retrospective analysis of mCRPC patients treated with sipuleucel-T and ongoing ADT alone. Within that group, we then identified a subset of sipuleucel-T-treated men with long-term disease control and no additional interventions. To independently confirm this finding, we evaluated a total of 336 patients from 4 large urology group practices treated with sipuleucel-T between 2010 and 2014 and identified 44 patients who met the same criteria and demonstrated evidence of PSA stabilization post sipuleucel-T treatment.

**Results:**

For this subgroup of patients, 79% (95% CI: 64.5%, 88.1%) survived 36 months with a median time to subsequent therapy of 17.8 months (95% CI 10.3, 25.3).

**Conclusions:**

Although patient selection could account for some or all of these results, these data support the utilization of sipuleucel-T alone in select mCRPC patients that is associated with a delay in disease progression and a good overall prognosis.

## Introduction

Sipuleucel-T is a therapeutic autologous cellular immunotherapy, produced by ex-vivo incubation of the patient’s own antigen presenting cells (APC’s) that have been activated with a recombinant fusion protein of prostatic acid phosphatase linked to an immune cell activator, granulocyte–macrophage colony-stimulating factor. Sipuleucel-T is the only FDA-approved anti-cancer vaccine for the specific treatment of men with mCRPC, based on a pivotal Phase III trial IMPACT, demonstrating an OS benefit with sipuleucel-T compared with placebo (median OS: 25.8 vs. 21.7 months, HR 0.78, 95% CI 0.61–0.98) [1]. Recent data support a greater relative and absolute improvement in overall survival in these men with mCRPC treated with sipuleucel-T based on lower pre-treatment PSA levels [1]. Despite this improvement in survival, evidence of PSA response, defined as reductions of at least 50% on two visits at least 4 weeks apart, were observed in only 2.6% of patients treated with sipuleucel-T in the IMPACT trial. Radiographic responses were also rare; a partial objective radiographic response was observed in only one patient (0.3%) in the sipuleucel-T group in the IMPACT study [1–3].

Since its approval in 2010, sipuleucel-T has been under-utilized for the treatment of mCRPC. The reasons may include its unique mechanism of action, its inherent expense/cost density, and the lack of PSA or radiographic response in clinical trials. In 2009, approximately 17,000 men in the US were diagnosed with asymptomatic or minimally symptomatic chemotherapy-naïve mCRPC, representing the patient population eligible for sipuleucel-T. This number is projected to grow to approximately 20,000 by the year 2020 [2]. However, only approximately 4000 patients are treated with sipuleucel-T on label each year (Dendreon Data On File 2017).

Anecdotally, we have observed improvement in serum PSA levels in several patients in our clinic, following treatment with sipuleucel-T alone. This raised the question of whether there is a patient population who achieve a PSA decline or delay in PSA progression following sipuleucel-T alone and is this associated with a longer survival benefit? To interrogate this question, we performed a retrospective review of patients treated with sipuleucel-T as monotherapy at our institution and based on this preliminary data, developed and conducted a multicenter validation study to confirm our findings in an independent cohort.

## Materials and methods

### Study design

This was a multi-center retrospective chart review of patients with mCRPC treated with standard-of-care sipuleucel-T between April 30, 2010 and July 31, 2014. Because there is no standard definition of PSA stabilization we define it below. Non-progressors included men with mCRPC who experienced PSA stabilization (PSA change +/− 20% of pre-treatment PSA value) for at least 2 months following completion of all 3 doses of sipuleucel-T treatment and prior to starting subsequent therapy. At least 6 months of available follow up data was required for inclusion. Concurrent LHRH agonist, LHRH antagonist, first generation anti-androgens (e.g., bicalutamide), bisphosphonates (e.g., zoledronic acid), and RANK ligand inhibitors (i.e., denosumab) were permitted; other concurrent prostate cancer therapies were not allowed. The Duke Cancer Institute cohort was followed until July 25, 2018 (data lock); subsequently, an independent external validation cohort was enrolled at four additional US Large Urology Group Practice Association (LUGPA) sites: Carolina Urologic Research Center (Myrtle Beach), Chesapeake Urology, The Urology Group (Cincinnati), and Urology Associates (Tennessee). The study was approved by the institutional review boards at each study center. The survival data was locked on March 8, 2018.

### Statistics

The primary outcome of this retrospective study was to identify and describe patients with PSA stabilization or decline following treatment with sipuleucel-T. The Duke cohort was used to explore and the LUGPA cohort was used to corroborate these findings. All men with mCRPC that received 3 doses of sipuleucel-T and experienced PSA stabilization or decline following treatment were included. Overall survival (OS) and time to subsequent therapy were estimated using Kaplan-Meier methods. OS was calculated from initiation of sipuleucel-T treatment to the date of death or last contact if censored. Estimates with 95% confidence intervals were generated for median OS, as well as 12-month, 24-month, and 36-month OS. Given the descriptive nature of this chart review, no formal sample size calculations were required.

### Patients

Patient cases were identified retrospectively from the treatment records of all patients receiving standard of care sipuleucel-T therapy with adequate follow-up at the individual centers inclusive of the study dates (April 30 2010 to July 31 2014). Patient records were reviewed and the cases which met our criteria for PSA stabilization, were identified. The study was reviewed as consent exempt at each center. We collected PSA results longitudinally following sipuleucel-T therapy at 2–3 month intervals. Patient records were assessed for the frequency of PSA stabilization and decline post-sipuleucel-T therapy, time to subsequent systemic treatment, treatment patterns of response following sipuleucel-T therapy, and overall survival at 1, 2, and 3 years post-treatment.

## Results

### Duke cohort

We identified a total of 94 patients that received sipuleucel-T between April 30, 2010 and July 31, 2014 with at least 6 months of follow-up. Of these, 4 patients (4.2%) met our criteria as being free of PSA progression for at least 2 months with no additional therapy. Interestingly while 23 out of 94 (24%) Duke patients were African American, 2 of the 4 (50 %) patients who met our criteria for PSA progression-free survival were African American (Table 1). In addition, demographics data on all of the patients who did not meet the criteria for this study are now presented in Supplemental Table 1. Based on these data the larger LUGPA cohort was evaluated.

**Table 1:** Baseline demographic and clinical characteristics of the Duke and LUGPA cohort patients

	Duke Pilot cohort (*n* = 4)	LUGPA PSA stabilization cohort (*n* = 44)
Age (years)		
Median	62	75
Range	55–78	56–95
Race no. (%)		
White	2 (50)	31 (59.7)
Black	2 (50)	13 (27.1)
Other	0 (0)	0 (0)
Unknown	0 (0)	0 (0)
Gleason sum no. (%)		
Missing	0 (0)	5 (11.4)
6	1 (25)	8 (18.2)
7	2 (50)	12 (27.3)
8–10	1 (25)	19 (43.2)
Prostatectomy at diagnosis no. (%)	1 (25)	14 (31.8)
Radiation at diagnosis no. (%)	2 (50)	12 (27.3)
Brachytherapy at diagnosis no. (%)	1 (25)	7 (15.9)
Cryotherapy at diagnosis no. (%)	0 (0)	1 (2.3)
Radiation at recurrence no. (%)	1 (25)	11 (25)
Cryotherapy at recurrence no. (%)	1 (25)	3 (6.8
ECOG performance status at initiation of sipuleucel-T no. (%)		
0	1 (25)	24 (54.6)
1	1 (25)	15 (34.1)
Unknown	2 (50)	5 (11.3)
Median PSA level at initiation of sipuleucel-T (ng/ml) (range)	9.3 (3.7–1111.2)	8.39 (0.05–540.3)
Sites of metastatic disease at initiation of sipuleucel-T – no. (%)		
Lymph node	3 (75)	8 (18.2)
Bone	0 (0)	32 (72.7)
Visceral	0 (0)	1 (2.3)
Missing	1 (20)	2 (4.5)
Concurrent first generation antiandrogen at Initiation of sipuleucel-T- No. (%)	2 (50)	8 (18.2)

### LUGPA Cohort (validation)

From 4 independent urology practices, we identified a total of 336 patients who received sipuleucel-T between April 30, 2010 and July 31, 2014 with at least 6 months of follow-up. Forty-four of these patients (13.1%) met our criteria for PSA stabilization. Demographic and clinical characteristics in the LUGPA cohort are shown in Table 1. 43.2% (*n* = 19) patients had a Gleason Score of 8 and above and 31.8% (*n* = 14) had undergone prostatectomy while 27.3% (*n* = 12) had radiation therapy and only 6.8% (*n* = 3) had cryotherapy. Fifty five percent of patients (*n* = 24) had an ECOG of 0. The median PSA prior to sipuleucel-T was 8.39 ng/ml. Seventy-three percent (*n* = 32) of patients had bone metastases, 18% (*n* = 8) had lymph node metastases and 1 patient had lung metastases (Table 1), at study entry.

### PSA stabilization and decline following treatment with Sipuleucel-T alone

PSA stabilization or decline 2 to 7 months following completion of sipuleucel-T was observed in 13.1% (*n* = 44 of 336) of patients in the LUGPA validation cohort. Six patients had not received subsequent therapy at the time of data lock. Among the 38 patients that received subsequent therapy following sipuleucel-T, the median time from the start of sipuleucel-T to subsequent therapy was 17.1 months (range 1.64 to 52.6 months). However, most patients demonstrated PSA rise much sooner than their initiation of subsequent therapy. For instance, 28 patients (63.6%) demonstrated a persistent decline or stabilization in PSA values at 4 months, but by 6 months only 18 patients (40.9%) had persistent PSA decline or stabilization, and only 12 (27.3%) by 9 months.

**Fig. 1:**
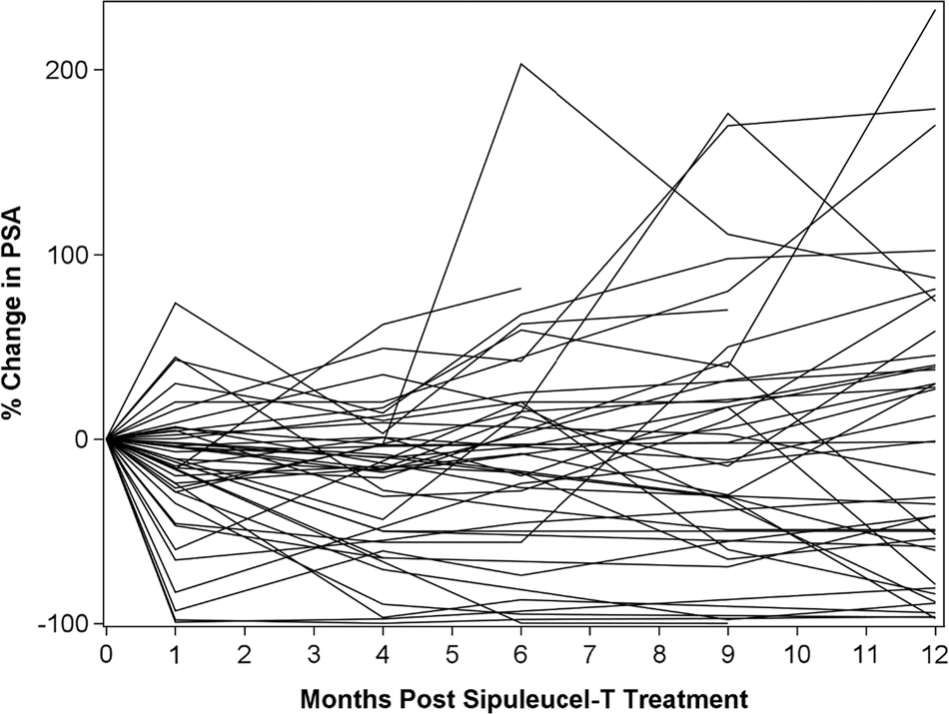
SPIDER plot of PSA baseline and subsequent change by case (LUGPA cohort):-*Y*-axis represents percent change in PSA compared to baseline (% change = 0 at month = 0). *X*-axis represents time in months from the initiation of sipuleucel-T. Each line represents the PSA curve for one patient post sipuleucel-T. Majority of the PSA curves show PSA stabilization or decline. Few PSAs which showed an initial rise, subsequently reached PSA stabilization in the absence of any additional therapy. Majority of these curves show PSA stabilization beyond 12 months in the absence of addition of any subsequent therapy. The curve continues for 12 months or until initiation of another therapy

Interestingly, we observed a range of PSA patterns following sipuleucel-T therapy in LUGPA cohort prior to going onto subsequent therapy (Fig. 1). The spider plot shows that most patients demonstrate some degree of PSA decline from baseline with several outliers demonstrating prolonged and dramatic PSA declines with no subsequent therapy. For instance, one patient did not receive subsequent therapy for 3-years following sipuleucel-T, three patients did not receive subsequent therapy for 4-years following sipuleucel-T, and one patient did not receive subsequent therapy for 5-years following sipuleucel-T.

### Overall survival

**Fig. 2:**
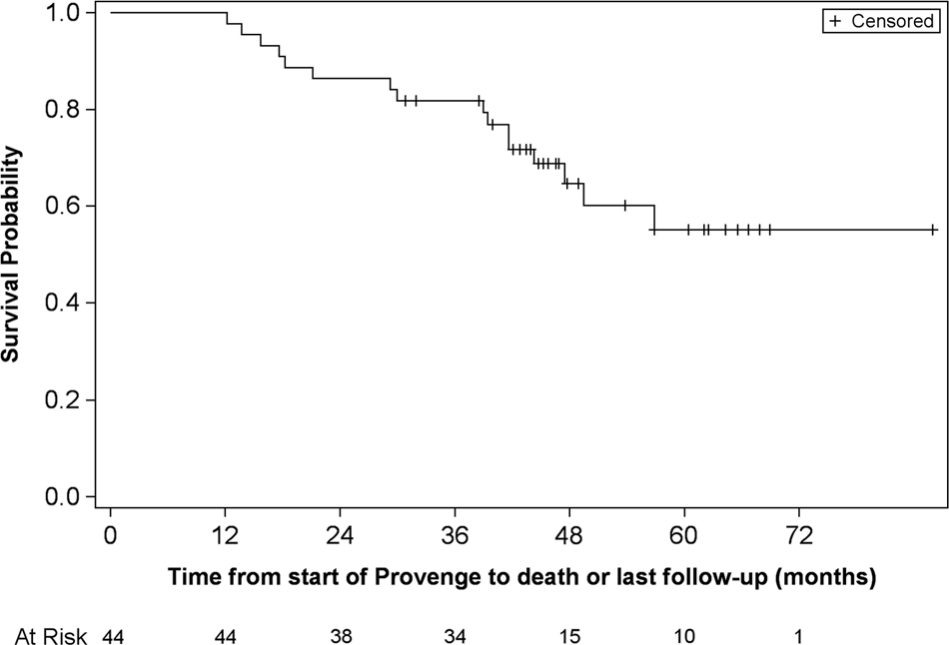
Kaplan–Meier plot of overall survival for enrolled patients (in months) (LUGPA cohort):-*Y*-axis represents the probability of patients alive while the *x*-axis represents time in months for 44 patients identified at 4 LUGPA sites. On the bottom of the figure are listed the number of patients at risk by time point. The median overall survival for this population has not been reached. Median follow up for this cohort was 96 months

Overall survival was evaluated for eligible patients in the LUGPA cohort (*n* = 44) and is depicted in the Kaplan-Meier plot (Fig. 2). Overall, 79% of patients were alive at 36-months post sipuleucel-T initiation. demonstrating a favorable prognosis despite no further delay in subsequent therapy.

### Race-based differences in PSA response

In the Duke cohort, 24% patients were African American, but 50% patients with delayed PSA progression were African American (2 out of 4). We observed similar favorable rates of PSA stabilization/response among Afican Americans in the LUGPA cohort as well. Specifically, 68 of 336 (20.2%) patients in the overall LUGPA cohort were African American compared to 13 of 44 (29.5%) of those who met our study criteria for PSA progression-free survival.

## Discussion

Sipuleucel-T is the first and only autologous cellular vaccine that has received FDA approval as an immunotherapy for patients with mCRPC based upon an overall survival benefit. However, based in part on its mechanism of action, it was not surprising that no immediate effects on PSA were observed and in general patients demonstrated relatively rapid PSA and radiographic progression on study [2–4]. We performed a retrospective cohort analysis in order to identify and describe patients with subsequent disease stabilization following sipuleucel-T alone. We focused on patients treated before July 2014 when abiraterone became widely available for chemotherapy-naïve patients. Over this timeframe we identified 13% of patients who exhibited PSA stabilization. We saw a trend of a greater percentage of African American patients meeting this criteria. This racial trend is interesting because a greater benefit to sipuleucel-T was seen in the African American cohort of the pivotal IMPACT study [1]. Furthermore, Sartor et al. presented data from the PROCEED registry, a large prospective database of mCRPC patients treated with sipuleucel-T in clinical practice, and demonstrated in a case-controlled analysis balanced for baseline PSA, a greater overall survival in African American patients compared to Caucasians [5]. These results support the hypothesis that host factors, associated with African American race, could positively impact the clinical benefit of sipuleucel-T. Given the small sample size of African American patients, we do not directly compare effects of sipuleucel-T on PSA levels in this particular patient population.

Our study has several important limitations. First and foremost, we cannot determine how much the natural history of prostate cancer could account for these findings. While disease stabilization is uncommon without a therapeutic intervention, it is possible that some or even most of this effect could be a result of patient selection. Our cohort may simply reflect a better prognosis subset of men with mCRPC undergoing standard of care sipuleucel T who have slow PSA kinetics. For context, the PREVAIL study comparing enzalutamide to placebo, included many patients in this disease setting. The placebo arm of PREVAIL demonstrated a 12-month rPFS of 12% and a 12-month PSA PFS of <5% [6]. These results would suggest that without any intervention, prolonged disease and PSA stabilization is uncommon. Regardless of whether this effect is due to patient selection or treatment effect, it does support the practice, in selected patients, of treating men with sipuleucel-T alone and following them even through subsequent PSA rise if this is not accompanied with other signs of progression (radiographic or clinical). We are unable to establish a cutoff point at which benefit is expected post therapy; however, our data support a potential clinical course following sipuleucel-T alone that can be indolent and associated with prolonged survival. It is important to note that although bicalutamide was given to 9 of the patients in our cohort, this was stopped at least 5 months prior to sipuleucel-T therapy. Only two of the pateints remained on bicalutamide during the treatment, thus suggesting that changes in PSA were not due to bicalutamide withdrawal.

Second, since our study was retrospective we are unable to determine significant immunological changes associated with a sustained decline in patients who did not progress. We are currently working towards establishing a relationship between PSA changes in patients post sipuleucel-T and their immune cell profiling and activation. Despite these limitations, the findings from this study support the hypothesis that a subset of patients exists in which sipuleucel-T alone can result in prolonged PSA stabilization which may lead to extended overall survival without any immediate additional intervention. In review of an earlier randomized trial of sipuleucel-T vs. placebo, a separation in PFS was seen in the bottom 30% of patients in favor of sipuleucel-T further suggesting a more proximal treatment effect in a subset of patients [2].

These findings are clinically relevant on several fronts. First, these data suggest that outlier or exceptional responders could exist following sipulecuel-T therapy and warrant further study. Second, these results support the delayed use of secondary hormonal therapies in patients immediately following sipuleucel-T treatment. Third, it is possible that future biomarkers or other clinical indicators could enrich for identification of these non-progressors either before or after treatment. Further validation of these findings in the PROCEED registry is underway and could further strengthen these hypotheses.

## Supplementary information

Figure S1

Figure S2

Table S1

## References

[1] Schellhammer PF, Chodak G, Whitmore JB, Sims R, Frohlich MW, Kantoff PW (2013). Lower baseline prostate-specific antigen is associated with a greater overall survival benefit from sipuleucel-T in the Immunotherapy for Prostate Adenocarcinoma Treatment (IMPACT) trial. Urology.

[2] Small EJ, Schellhammer PF, Higano CS, Redfern CH, Nemunaitis JJ, Valone FH (2006). Placebo-controlled phase III trial of immunologic therapy with sipuleucel-T (APC8015) in patients with metastatic, asymptomatic hormone refractory prostate cancer. J Clin Oncol.

[3] Higano CS, Schellhammer PF, Small EJ, Burch PA, Nemunaitis J, Yuh L (2009). Integrated data from 2 randomized, double-blind, placebo-controlled, phase 3 trials of active cellular immunotherapy with sipuleucel-T in advanced prostate cancer. Cancer.

[4] Kantoff PW, Higano CS, Shore ND, Berger ER, Small EJ, Penson DF (2010). Sipuleucel-T immunotherapy for castration-resistant prostate cancer. N Engl J Med.

[5] Oliver A, Sartor AA, Ahaghotu Chiledum, McLeod David, Cooperberg Matthew, Penson David (2017). Overall survival analysis of african american and caucasian patients receiving sipuleucel-T: preliminary data from the proceed registry. J Urol.

[6] Beer TM, Armstrong AJ, Rathkopf DE, Loriot Y, Sternberg CN, Higano CS (2014). Enzalutamide in metastatic prostate cancer before chemotherapy. N Engl J Med.

