# Supplementary material for: Prolonged PSA stabilization and overall survival following sipuleucel-T monotherapy in metastatic castration-resistant prostate cancer patients
Source: Prostate Cancer Prostatic Dis. 2019 Apr 12;22(4):588–92. doi: 10.1038/s41391-019-0144-3 (PMC6853838; doi:10.1038/s41391-019-0144-3)

Supplemental Figure 1: OS for Duke patients. A: Kaplan-Meier plot of overall survival calculated from the start of Sipuleucel-T to death or last follow-up in the Duke cohort.

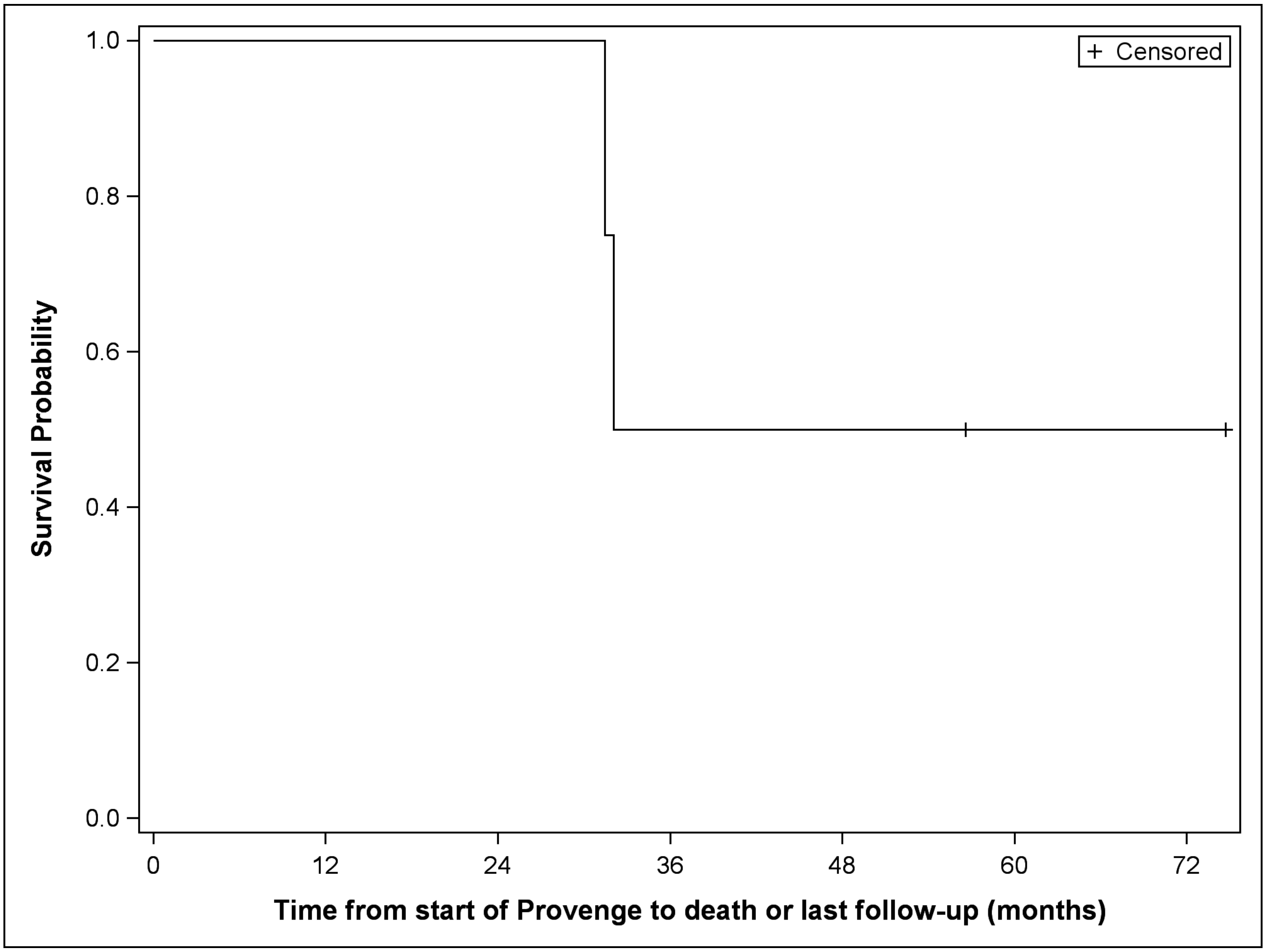

Supplement: Supplementary file 1 — Figure S1 [file 41391_2019_144_MOESM1_ESM.pdf]
