# Supplementary material for: Prolonged PSA stabilization and overall survival following sipuleucel-T monotherapy in metastatic castration-resistant prostate cancer patients
Source: Prostate Cancer Prostatic Dis. 2019 Apr 12;22(4):588–92. doi: 10.1038/s41391-019-0144-3 (PMC6853838; doi:10.1038/s41391-019-0144-3)
Supplement: Supplementary file 2 — Figure S2 [file 41391_2019_144_MOESM2_ESM.pdf]

Supplemental Figure 2: Time to subsequent therapy. Kaplan-Meier plot of overall survival calculated from the start of Sipuleucel-T to time of subsequent therapy or last follow-up. Median time to subsequent therapy was 17.8 months (95% CI: 10.5, 25.3).

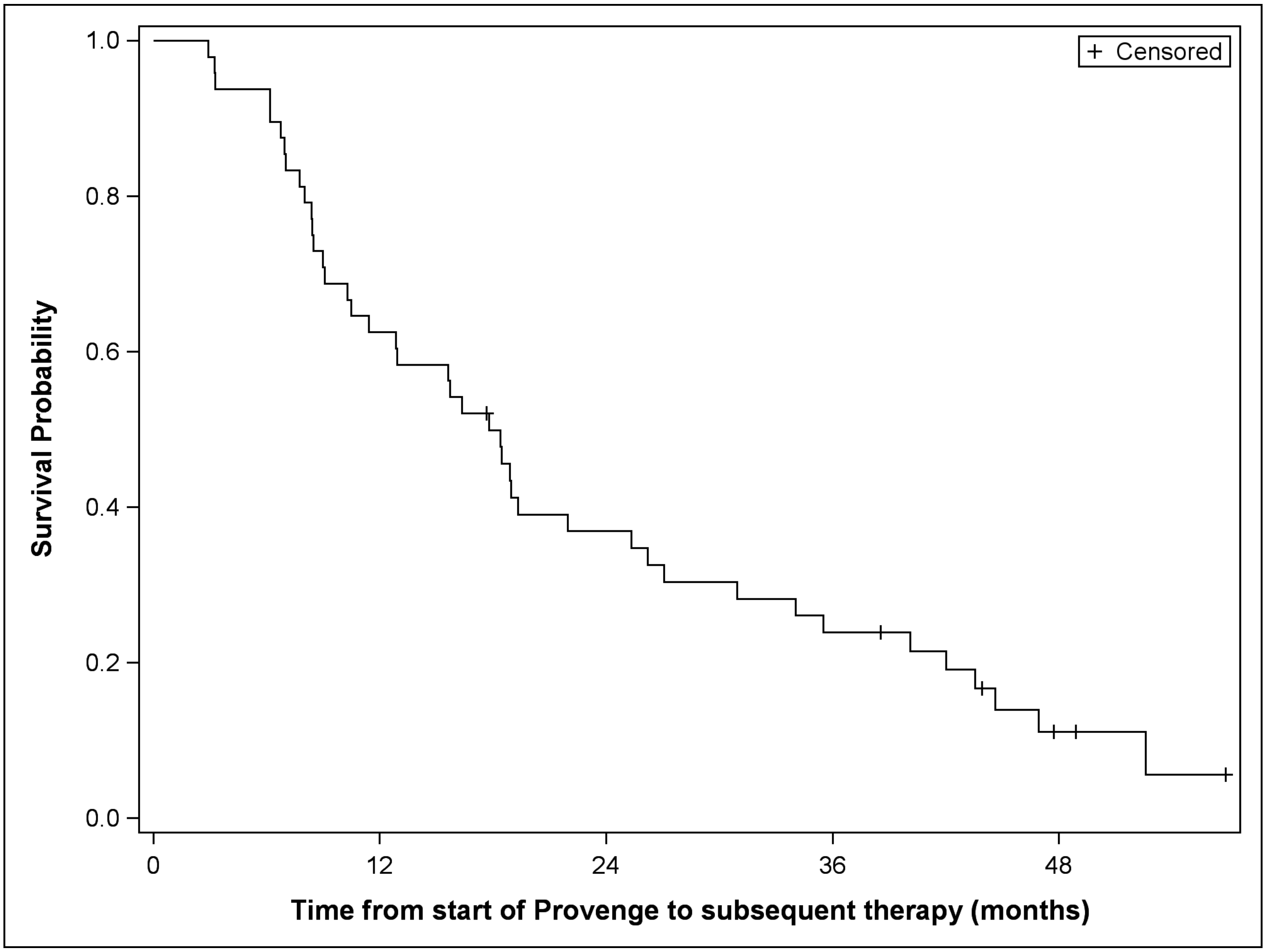

Median time to subsequent therapy: 17.8 months (95% CI: 10.5, 25.3)
