# Supplementary material for: Prolonged PSA stabilization and overall survival following sipuleucel-T monotherapy in metastatic castration-resistant prostate cancer patients
Source: Prostate Cancer Prostatic Dis. 2019 Apr 12;22(4):588–92. doi: 10.1038/s41391-019-0144-3 (PMC6853838; doi:10.1038/s41391-019-0144-3)
Supplement: Supplementary file 3 — Table S1 [file 41391_2019_144_MOESM3_ESM.docx]

|  | **N** | **%** |
| --- | --- | --- |
| **Primary Gleason** | 11 | 12.22 |
| Missing |  |  |
| 3 | 22 | 24.44 |
| 4 | 47 | 52.22 |
| 5 | 10 | 11.11 |
| **Secondary Gleason** | 11 | 12.22 |
| Missing |  |  |
| 2 | 1 | 1.11 |
| 3 | 28 | 31.11 |
| 4 | 33 | 36.67 |
| 5 | 17 | 18.89 |
| **Gleason score** | 8 | 8.89 |
| Missing |  |  |
| 5 | 1 | 1.11 |
| 6 | 12 | 13.33 |
| 7 | 27 | 30.00 |
| 8 | 17 | 18.89 |
| 9 | 23 | 25.56 |
| 10 | 2 | 2.22 |
| **Local Procedures at diagnosis: Prostatecomy** | 52 | 57.78 |
| No |  |  |
| Yes | 38 | 42.22 |
| **Local Procedures at diagnosis: Radiation Therapy** | 46 | 51.11 |
| No |  |  |
| Yes | 44 | 48.89 |
| **Local Procedures at diagnosis: Brachytherapy** | 83 | 92.22 |
| No |  |  |
| Yes | 7 | 7.78 |
| **Local Procedures at diagnosis: Cyrotherapy** | 89 | 98.89 |
| No |  |  |
| Yes | 1 | 1.11 |
| **Local Procedures at diagnosis: Orchietecomy** | 89 | 98.89 |
| No |  |  |
| Yes | 1 | 1.11 |
| **Local Procedures at recurrence: Prostatecomy** | 90 | 100.00 |
| No |  |  |
| **Local Procedures at recurrence: Radiation Therapy** | 61 | 67.78 |
| No |  |  |
| Yes | 29 | 32.22 |
| **Local Procedures at recurrence: Brachytherapy** | 90 | 100.00 |
| No |  |  |
| **Local Procedures at recurrence: Cyrotherapy** | 88 | 97.78 |
| No |  |  |
| Yes | 2 | 2.22 |
| **Local Procedures at recurrence: Orchietecomy** | 90 | 100.00 |
| No |  |  |
| **Metastatic Sites: Bone** | 17 | 18.89 |
| No |  |  |
| Yes | 73 | 81.11 |
| **Metastatic Sites: Lymph node** | 58 | 64.44 |
| No |  |  |
| Yes | 32 | 35.56 |
| **Metastatic Sites: Lung** | 85 | 94.44 |
| No |  |  |
| Yes | 5 | 5.56 |
| **Other metastatic site** | 88 | 97.78 |
| N/A |  |  |
| Biopsy-proven prostate cancer invading rectum | 1 | 1.11 |
| Left temporal calvarium met | 1 | 1.11 |
| **HIV/AIDS** | 90 | 100.00 |
| Absent / Not Found |  |  |
| **Hepatitis B/C** | 90 | 100.00 |
| Absent / Not Found |  |  |
| **Cerebrovascular disease (any history of, including TIA** | 87 | 96.67 |
| Absent / Not Found |  |  |
| Present | 3 | 3.33 |
| **Chronic pulmonary disease** | 90 | 100.00 |
| Absent / Not Found |  |  |
| **Congestive heart failure (history of treatment for** | 89 | 98.89 |
| Absent / Not Found |  |  |
| Present | 1 | 1.11 |
| **Myocardial infarction (including history of** | 86 | 95.56 |
| Absent / Not Found |  |  |
| Present | 4 | 4.44 |
| **Dementia** | 89 | 98.89 |
| Absent / Not Found |  |  |
| Present | 1 | 1.11 |
| **Hypertension** | 39 | 43.33 |
| Absent / Not Found |  |  |
| Present | 51 | 56.67 |
| **Hyperthyroidism** | 90 | 100.00 |
| Absent / Not Found |  |  |
| **Hypothyroidism** | 85 | 94.44 |
| Absent / Not Found |  |  |
| Present | 5 | 5.56 |
| **Peptic Ulcer Disease** | 1 | 1.11 |
| Missing |  |  |
| Absent / Not Found | 88 | 97.78 |
| Present | 1 | 1.11 |
| **Chronic Liver Disease** | 90 | 100.00 |
| Absent / Not Found |  |  |
| **Chronic Kidney Disease** | 2 | 2.22 |
| Missing |  |  |
| Absent / Not Found | 81 | 90.00 |
| Present | 7 | 7.78 |
| **Diabetes** | 70 | 77.78 |
| Absent / Not Found |  |  |
| Present | 20 | 22.22 |
| **History of Drug Abuse** | 2 | 2.22 |
| Missing |  |  |
| Absent / Not Found | 88 | 97.78 |
| **History of Alcohol Abuse** | 2 | 2.22 |
| Missing |  |  |
| Absent / Not Found | 88 | 97.78 |
| **Hyperlipidemia** | 1 | 1.11 |
| Missing |  |  |
| Absent / Not Found | 51 | 56.67 |
| Present | 38 | 42.22 |
| **History of DVT or PE** | 1 | 1.11 |
| Missing |  |  |
| Absent / Not Found | 85 | 94.44 |
| Present | 4 | 4.44 |
| **Malignant solid tumor (other than PCa** | 85 | 94.44 |
| Absent / Not found |  |  |
| Non-metastatic | 5 | 5.56 |
| **Smoking status** | 48 | 53.33 |
| Never Smoked |  |  |
| Current | 4 | 4.44 |
| Past | 33 | 36.67 |
| Unknown | 5 | 5.56 |
| **ECOG Value at Initiation of Provenge (+/- 1 month** | 20 | 22.22 |
| 0 |  |  |
| 1 | 10 | 11.11 |
| 2 | 2 | 2.22 |
| Unknown / Not documented | 58 | 64.44 |
| **Karnofsky Value** | 37 | 41.11 |
| Missing |  |  |
| 80 | 3 | 3.33 |
| 90 | 23 | 25.56 |
| 100 | 27 | 30.00 |
| **Total** | 90 | 100.00 |

|  | **N** | **Min** | **Median** | **Max** |
| --- | --- | --- | --- | --- |
| **PSA value at Initial Diagnosis of PCa** | 73 | 0.80 | 12.99 | 1285.00 |
| **PSA at CRPC** | 86 | 0.10 | 3.89 | 82.00 |
| **PSA at Metastasis** | 78 | 0.20 | 20.75 | 1285.00 |
| **Most recent PSA value prior to Provenge** | 86 | 0.10 | 10.95 | 2862.40 |
